# Sensitivity of Physiological Measures of Acute Driver Stress: A Meta-Analytic Review

**DOI:** 10.3389/fnrgo.2021.756473

**Published:** 2021-12-14

**Authors:** Laora Kerautret, Stephanie Dabic, Jordan Navarro

**Affiliations:** ^1^Laboratoire d'Etude des Mécanismes Cognitifs, University Lyon 2, Lyon, France; ^2^Valeo Interior Controls, Annemasse, France; ^3^Institut Universitaire de France, Paris, France

**Keywords:** driver, stress, physiological, measures, sensitivity, individual, ambient, meta-analysis

## Abstract

**Background:** The link between driving performance impairment and driver stress is well-established. Identifying and understanding driver stress is therefore of major interest in terms of safety. Although many studies have examined various physiological measures to identify driver stress, none of these has as yet been definitively confirmed as offering definitive all-round validity in practice.

**Aims:** Based on the data available in the literature, our main goal was to provide a quantitative assessment of the sensitivity of the physiological measures used to identify driver stress. The secondary goal was to assess the influence of individual factors (i.e., characteristics of the driver) and ambient factors (i.e., characteristics of the context) on driver stress. Age and gender were investigated as individual factors. Ambient factors were considered through the experimental apparatus (real-road vs. driving simulator), automation driving (manual driving vs. fully autonomous driving) and stressor exposure duration (short vs. long-term).

**Method:** Nine meta-analyses were conducted to quantify the changes in each physiological measure during high-stress vs. low-stress driving. Meta-regressions and subgroup analyses were performed to assess the moderating effect of individual and ambient factors on driver stress.

**Results:** Changes in stress responses suggest that several measures are sensitive to levels of driver stress, including heart rate, R-R intervals (RRI) and pupil diameter. No influence of individual and ambient factors was observed for heart rate.

**Applications and Perspective:** These results provide an initial guide to researchers and practitioners when selecting physiological measures for quantifying driver stress. Based on the results, it is recommended that future research and practice use (*i*) multiple physiological measures, (*ii*) a triangulation-based methodology (combination of measurement modalities), and (*iii*) a multifactorial approach (analysis of the interaction of stressors and moderators).

## Introduction

### Identifying Driver Stress: A Safety and Comfort Challenge

Driving is a complex activity that takes place in a dynamic environment where safety critical situations abound. Therefore, many driving situations can lead the driver to experience stress, such as bad weather, low visibility, complex driver-environment interactions, and particular driving routes (Hill and Boyle, 2007; Rodrigues et al., 2015; Rastgoo et al., 2018). Although driver stress can be experienced as positive (i.e., eustress), the focus here is placed on its negative dimension (i.e., distress), which is more critical for well-being and road safety (Chung et al., 2019). Associated with negative emotions (e.g., anxiety, Kontogiannis, 2006, fear, Schmidt-Daffy, 2013, anger, Emo et al., 2016; Ooi et al., 2018; Gotardi et al., 2019) and the subjective feeling that the situation exceeds the individual's coping abilities (Selye, 1976), distress can lead to poor driving performances and risky behaviors (Matthews et al., 1998; Hancock and Desmond, 2001; Ge et al., 2014; Rendon-Velez et al., 2016). Given the causal relationship between distress and poor driving performance, finding measures that are sensitive to the level of stress is crucial if we are to gain a better understanding of this disturbed state and develop future remediation and support strategies.

Driver stress has often been identified on the basis of various subjective scales, including the Driver Stress Inventory (Matthews et al., 1997) and Driver Behavior Inventory (Gulian et al., 1989; Glendon et al., 1993). Although these scales have proven useful for capturing the multifaceted nature of driver stress, they may also be limited by individuals' inaccuracy in self-reporting stress levels. What is more, relationships with the neuroticism dimension have been shown to account for some of the inaccuracy of subjective stress ratings (McCrae, 1990; Espejo et al., 2011). Driver stress has also been inferred to a large extent from the analysis of driving behaviors, such as steering wheel motion, speed, acceleration, braking, overtaking, and lane keeping (Schießl, 2008; Rigas et al., 2012; Lanatà et al., 2014; Miller and Boyle, 2015; Rendon-Velez et al., 2016; Lee et al., 2017). Again, this method of identifying driver stress has some disadvantages. In addition to being a discontinuous stress measure, it can also be problematic in the context of automated driving since the driver is intended to be replaced by automation, leading to a decrease in driving behaviors (Lohani et al., 2019). Unlike subjective assessments and analysis of specific driving behaviors, physiological measures offer empirical evidence—objective and continuous—of the stress response (Plarre et al., 2011). Physiological measures thus offer a direct insight into the psychological and physiological adaptability of individuals dealing with stressful situations (Hancock and Warm, 1989). Finally, physiological measures remain relevant for monitoring driver stress during highly automated driving, during which drivers are not continuously in physical control of the vehicle.

Historically, stress responses have been compared to alarm states of the body, triggered by physical threats from the environment and intended to prepare the body for action (Selye, 1956). The alarm analogy provides a clear way of understanding the role of the physiological mechanisms that underlie stress responses and facilitate fast action-oriented reactions. Functionally, these mechanisms reflect a coactivation of autonomic components resulting in sympathetic autonomic stimulation and parasympathetic autonomic withdrawal, thus minimizing a vagal “braking” action on the motor system (Roelofs, 2017). Among physiological responses, cardiac measures are generally favored by researchers and practitioners for quantifying stress states. The most commonly used measures to explore cardiac activity are heart rate and Heart Rate Variability (HRV) (Alberdi et al., 2016). While heart rate focuses on contraction frequency, HRV is a measure of the time that elapses between contractions. The analysis of the time series of beat-to-beat intervals provides additional information since it reflects the heart's ability to adapt to changes by detecting and responding to stimuli over time (Acharya et al., 2006; Kim H. G. et al., 2018). The idea is that an individual with a low variability between heartbeats in a stressful context would have a low capacity to deal with stressful stimuli. In a driving context, a cardiac response to stressful stimuli is usually observed through an increase in heart rate (Healey and Picard, 2005; Lee et al., 2007; Cottrell and Barton, 2012; Guo et al., 2013; Zhao et al., 2014; Reimer et al., 2016; Rendon-Velez et al., 2016; Magana and Munoz-Organero, 2017; Antoun et al., 2018; Haouij et al., 2018; Khattak et al., 2018; Gotardi et al., 2019; Heikoop et al., 2019; Meesit et al., 2020) and a decrease in HRV (Lee et al., 2007; Yu et al., 2016; Heikoop et al., 2017; Magana and Munoz-Organero, 2017; Antoun et al., 2018; Rastgoo et al., 2019; Tavakoli et al., 2020; Zhao et al., 2020). Other physiological responses have also been studied as indexes of driver stress levels, such as changes in electrodermal activity (Healey and Picard, 2005; Cottrell and Barton, 2012; Pedrotti et al., 2014; Eisel et al., 2016; Morris et al., 2017; Ooi et al., 2018; Paredes et al., 2018; Zontone et al., 2020, 2021), breathing (Healey and Picard, 2005; Rendon-Velez et al., 2016; Balters et al., 2018; Haouij et al., 2018; Napoletano and Rossi, 2018; Heikoop et al., 2019; Zhao et al., 2020), blood pressure (Yamakoshi et al., 2008; Antoun et al., 2018), skin temperature (Yamakoshi et al., 2007, 2008; Zhao et al., 2020), muscle activation (Healey and Picard, 2005; Morris et al., 2017), pupil diameter (Pedrotti et al., 2014; Rendon-Velez et al., 2016; Zontone et al., 2021) and electrical brain activity (Kim S. et al., 2018; Halim and Rehan, 2020). Despite the numerous physiological responses studied, none of them has been validated as a definitive measure for identifying driver stress. Therefore, the use of a measure is often guided by practical and experimental design constraints (for a review of the advantages and disadvantages of physiological measures for assessing cognitive states in lab and real-world driving, see Lohani et al., 2019). Nevertheless, we believe that it is necessary for researchers and practitioners to base their measure selection decisions on both the practical constraints and the sensitivity to identify driver stress. Measure sensitivity refers to a measure's ability to discriminate between two levels of a psychological state (e.g., high and low stress) (Hughes et al., 2019). To date, the sensitivity of the driver stress measure has not been directly evaluated. Therefore, there is a need to specifically study the sensitivity of each physiological measure to driver stress to assist researchers and practitioners in measure selection.

### Identifying Moderators of Driver Stress: A Theoretical Approach

Stress is a psycho-physiological state resulting from the influence of a stressor moderated by individual and ambient factors (Folkman and Lazarus, 1984; Matthews, 2002). In an automotive context, individual factors refer to the intrinsic characteristics of the driver (e.g., personality traits, demographic criteria), while ambient factors refer to the contextual effects (i.e., the circumstances in which a stressor operates).

Among the individual factors that may influence driver stress, age has probably been the most studied, particularly from a subjective perspective using self-report scales (Hartley and El Hassani, 1994; Simon and Corbett, 1996; Kloimüller et al., 2000). Despite these extensive investigations, the direction of the relationship between age and driver stress remains unclear. Indeed, some studies have found greater stress levels in older populations (Hill and Boyle, 2007) and explained this in terms of lower cognitive and physical abilities. Conversely, other studies have found lower stress levels in older populations (Langford and Glendon, 2002), which they have explained in part in terms of lower aggressiveness (Matthews et al., 1991; Westerman and Haigney, 2000) and more extensive driving experience (Gulian et al., 1990). Given the discrepancies at the subjective level, physiological measures provide objective ways of determining both the existence of the relationship and its direction. To our knowledge, only one study has found an effect of age on acute driver stress using physiological measures (Zhao et al., 2020). However, given the small number of participants included in this study (3 younger and 3 older), this effect deserves to be further explored. Like age, gender is an individual factor whose effect on driver stress is also debated. While some studies have found no effect of gender on driver stress using subjective scales (Wickens et al., 2015), others have reported higher stress levels in female drivers than male drivers based on cardiac (Guo et al., 2013) and hormone dosage measurements (Seeman et al., 1995).

In line with Hancock and Warm (1989), who recommended considering in stress studies both the demand imposed by the task and the type of environment, we suggest that automation (manual vs. autonomous) and stressor exposure duration (short vs. long-term) might be relevant factors when considering the driving task demand, while apparatus type (real vehicle vs. driving simulator) would make it possible to take account of the type of driving environment. We believe these three ambient factors to be of interest because they are either often debated in the literature (e.g., automation and apparatus), or have been the object of little direct study (e.g., stressor exposure duration).

#### Driving Automation

Interest in automated driving systems has grown over the last decade, in particular to compensate for the human errors in driving. More specifically in an automotive context, it is unclear whether a fully automated vehicle increases or reduces driver stress. Some authors have found positive effects of driving automation by reducing distress and enhancing driver attention (Funke et al., 2007), others have reported reduced driver stress coupled with a decrease in workload (Stanton and Young, 2005), while yet others have argued that autonomous driving increases driver stress due, in particular, to a lack of trust in the autonomous vehicle (Morris et al., 2017). Consequently, investigating this question would contribute to the development of automated driving systems adapted to the profiles of drivers and to given road situations.

#### Stressor Exposure Duration

The question regarding the existence of physiological differences between short and long periods of driving under acute stress has been little studied to date. A review of the literature came close to addressing this question by examining physiological responses to driver stress over short and long time periods (Antoun et al., 2017). However, due to the small number of studies collected, evidence of stress over a short time period was not revealed, thus reducing conclusions. The question therefore remains open.

#### Apparatus Type

With respect to the apparatus, the question of whether a driving simulator vs. a real vehicle is a valid way of studying internal driver states, such as stress, is unresolved. If the validity of simulators is confirmed, it is expected that observations made in a driving simulator will be equivalent to those made under real driving conditions. However, previous studies have reported contradictory results which make it difficult to draw clear conclusions. Taking the example of using mean heart rate to investigate validity, studies have shown a good level of correspondence between the simulator and the real road (Li et al., 2013). In contrast, other studies have found higher heart rates on real vehicles (Engström et al., 2005; Johnson et al., 2011). The fact that another study found both an absence of difference and a difference between the simulator and the real road depending on the driving situation, i.e., speed maintenance task and exposure to road hazards, respectively (Gemonet et al., 2021), further raises the question of the validity of the driving simulator for identifying driver stress in any driving situation.

### Aims

We undertook a meta-analysis of the existing literature investigating driver stress, first to address, at a practical level, the difficulty researchers and practitioners have in selecting physiological measures for quantifying driver stress, and second, to gain insights into the relationship between driver stress and its moderators. The objectives were three-fold: (*i*) to investigate the sensitivity of each physiological measure used to quantify driver stress, (*ii*) to assess the moderating effect of the population type on driver stress, and (*iii*) to identify whether driver stress is influenced by ambient effects in the environment in which the driving task takes place.

## Methods

### Search Strategy

This meta-analytical review was conducted in accordance with the Preferred Reporting Items for Systematic Reviews and Meta-Analyses (PRISMA) guidelines (Moher et al., 2009).

Two investigators searched for articles in the electronic database, Google Scholar. The only limitation in terms of date was publication prior to February 2021. The following search terms were used: “{[(driver OR driving) AND (stress OR distress)] OR [(car) AND (stress OR distress)]}.” These were then combined with additional terms related, first, to fields of research in which driver stress has been addressed: “psychological,” “physiological,” “behavior,” “detection,” “recognition” and, second, to the response of interest: “acute,” “response,” “change.” In addition, a snowballing approach (Wohlin, 2014) was used to retrieve additional references. Duplicate records were systematically removed.

**Figure 1:**
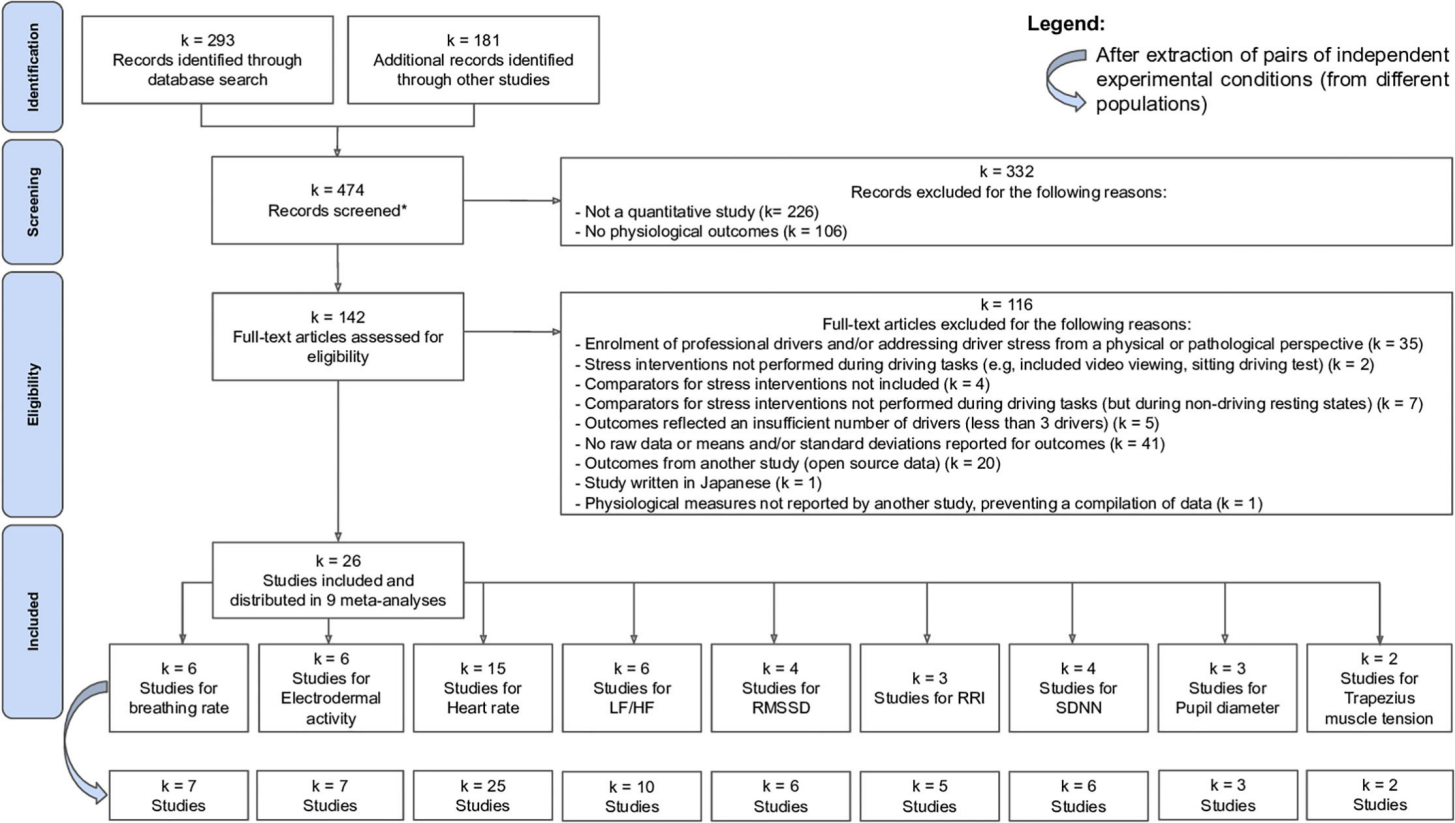
PRISMA flowchart describing the methodology and search results. LF/HF, ratio of low frequency to high frequency; RMSSD, root mean square of successive differences among successive R-R normal intervals; RRI, means of R-R intervals; SDNN, standard deviation of normal R-R intervals.

Each record was then screened (title, abstract and keywords) by the investigators in order to apply the eligibility criteria. The same procedure was carried out for the full-text articles. Any discrepancy between the investigators was resolved by discussion with a third investigator. The study selection process is described in Figure 1 (PRISMA diagram).

### Eligibility Criteria

We used the PICOS approach (Moher et al., 2015) to define the characteristics of studies eligible for inclusion in terms of population, interventions, comparators, outcomes and study design.

#### Population

Non-professional car drivers of all ages and genders, with no evidence of psychological or neurological disorders, were included.

#### Interventions

Stress interventions included driving tasks performed under high stress. Although the definition of “stress” or “high stress” is presumably a reflection of each author's particular standpoint, and the term has thus certainly been interpreted in many different ways, we decided to use Matthews' (2002) definition of driver stress to study similar stress interventions. Driver stress is thus interpreted as a psychological construct resulting from the stressful situation (involving stressors and ambient factors) and individual factors. Therefore, interventions in which driver stress was not a psychological construct but the product of physical action on the body were excluded. This was the case for stress interventions involving cold temperatures, pain, chronic illness, driving for long periods and monotonous driving periods.

#### Comparators

Comparators for the stress interventions were driving tasks performed under low stress.

#### Outcomes

All the included studies estimated driver stress based on physiological measures. All physiological outcomes were quantitatively reported as raw data or as means and standard deviations to allow the calculation of effect sizes. All physiological outcomes had been observed in at least three drivers.

#### Study Design

Only peer-reviewed quantitative physiological studies written in English were included in the analyses. All included studies contained a physiological measure also found in at least one other study to make it possible to compile the data required for a meta-analysis.

### Data Extraction

For each included study, two investigators independently extracted the following data: demographic variables (sample size, mean age and gender ratio), ambient variables (apparatus, driving automation and stressor exposure duration), stress interventions and comparators (i.e., pairwise comparisons including a high stress intervention vs. a low stress intervention), statistical indices for the stress interventions and comparators (means and standard deviations) and type of physiological measure used.

When data was missing, the corresponding authors were contacted and asked for additional data. The WebPlotDigitizer software (Rohatgi, 2014) was also used to extract numerical values from the plot when numerical means and/or standard deviations were not reported.

For each included scientific paper in which driver stress was assessed in multiple population groups (e.g., older and younger participants), each pairwise comparison belonging to a given group was treated as a separate and independent study. As a result, and for the sake of clarity, we will use the term “study” in the following sections to refer to a pairwise comparison into a given group and not to the scientific paper from which it was derived. In addition, in studies that reported multiple stress interventions in the same population, the various stress interventions were averaged when raw data was available. This precaution was taken to avoid introducing an error due to the non-processed correlation between the condition effects estimated from multiple comparisons (Higgins et al., 2011). If raw data was not available, the highest-stress intervention was retained and the others were excluded. Although the strategy for selecting interventions is less recommended than combining interventions, it is generally difficult to obtain the raw data from each study, as would be required in order to compute the overall mean and standard deviation.

### Meta-Analyses

Nine meta-analyses were conducted separately, one for each physiological measure. All analyses were carried out using JASP software (version 0.14.0.0). Due to different experimental designs and sample characteristics across included studies, we used random-effects models in an attempt to generalize our results beyond the studies included in our meta-analyses (Borenstein et al., 2010).

In keeping with previous studies that have tackled the issue of the sensitivity of physiological measures (Matthews et al., 2015; Hughes et al., 2019), we used effect size to determine the sensitivity of each measure of driver stress. Cohen's d effect size with 95% confidence intervals (95% CI) were first calculated for each study (i.e., for each pairwise comparison) based on the means, standard deviations and sample sizes (Cohen, 1988). Given the small sample sizes, Hedges' *g* was subsequently preferred to Cohen's *d* (Durlak, 2009). Hedges' *g* uses pooled weighted standard deviations instead of the pooled standard deviations used by Cohen's *d*. Mathematical equations used to compute effect size for each study are presented in the Supplementary Material 1. All effect sizes calculated for each study and corresponding to the same physiological measure were then aggregated to derive an overall summary effect size. A positive summary effect size indicated a positive effect of the stress intervention on all physiological measures except for HRV time-domain features (RRI, RMSSD and SDNN), for which a negative summary effect size suggested a positive effect of the stress intervention. Using Cohen's interpretation guidelines, the magnitude of the overall summary effect size was considered as small up to 0.2, medium up to 0.5, and large up to 0.8 (Cohen, 1988). The α level for significance was set at *p* < 0.05.

To quantify heterogeneity of the overall summary effect size, i.e., the inconsistency of effect sizes across a set of studies (Del Re, 2015), *Q*-statistic, *I*^2^-statistic and τ^2^ were explored. *Q*-statistic indicated the statistically significant presence of heterogeneity between effect sizes, *I*^2^-statistic estimated the proportion of heterogeneity (low if *I*^2^ = 25%, moderate if *I*^2^ = 50%, large if *I*^2^ = 75%), and τ^2^ referred to the absolute value of true variance across studies.

Publication bias was first assessed by visually inspecting the funnel plots. If an asymmetry was detected, a rank correlation test and an Egger's regression test (Egger et al., 1997) were run to assess the significance of the publication bias. Finally, the file drawer issue was assessed by Rosenthal's fail-safe N (Rosenthal, 1979). Fail-safe N refers to the number of studies that would have to be included in order to indicate that the stress intervention had no effect and that would be necessary for the meta-analysis to become non-significant. The file drawer problem was considered to be minor when the observed significance of fail-safe N was lower than the target significance level (*p* = 0.05), thus suggesting that the outcome of the meta-analysis was not affected by potential bias.

### Moderator Analyses

Moderator analyses were undertaken if each measure met the three eligibility criteria: (1) significant summary effect size, (2) significant heterogeneity in summary effect size and (3) sufficient number of available studies (*k* ≥ 5) to allow comparisons (Hughes et al., 2019). Meta-regressions were used when the factors studied were continuous variables, while subgroup analyses were conducted when the factors examined were categorical variables.

### Individual Factors

Age and gender—two individual factors—were investigated by running meta-regressions to assess their moderating effect on driver stress.

### Ambient Factors

The influence of three ambient factors on driver stress was studied by proceeding to subgroup analyses. These factors were: apparatus, driving automation and stressor exposure duration. In order to study the effect of apparatus type, a first subgroup was formed by pooling studies performed in a real vehicle while a second subgroup included studies performed in a driving simulator. Two independent analyses were then run to compute a summary effect for each subgroup. Finally, we analyzed whether the two summary effect sizes differed significantly, first by looking for overlaps between their confidence intervals and second by using a Wald-type test. The same procedure was repeated to explore driving automation and thus compare studies conducted in manual driving (first subgroup) and in fully autonomous driving (second subgroup). Again, the same procedure was used to investigate stressor exposure duration by comparing studies involving short-term exposure (first subgroup) and long-term exposure (second subgroup). The subgroups were formed by arbitrarily setting a threshold at 10 mins so that exposure times below the threshold comprised the first subgroup and exposure times above the threshold comprised the second subgroup.

## Results

### Search Results

The primary search yielded 474 records. After screening each record, 332 abstracts were excluded in line with the eligibility criteria. The remaining 142 studies were then assessed for eligibility based on full-length articles. Finally, 26 references were included and distributed across 9 meta-analyses to permit independent exploration of 9 physiological measures (Healey and Picard, 2005; Schießl, 2008; Cottrell and Barton, 2012; Miller and Boyle, 2013; Manseer and Riener, 2014; Pedrotti et al., 2014; Zhao et al., 2014, 2020; Chen, 2015; Rendon-Velez et al., 2016; Yu et al., 2016; Heikoop et al., 2017, 2019; Magana and Munoz-Organero, 2017; Morris et al., 2017; Haouij et al., 2018; Khattak et al., 2018; Napoletano and Rossi, 2018; Ooi et al., 2018; Paredes et al., 2018; Gotardi et al., 2019; Rastgoo et al., 2019; Meesit et al., 2020; Tavakoli et al., 2020; Zontone et al., 2020, 2021) (Figure 1).

### Characteristics of Studies

A qualitative review of the literature indicated that driver stress was indexed by breathing rate in 7 studies (156 drivers), electrodermal activity in 7 studies (187 drivers), heart rate in 25 studies (501 drivers), the ratio of Low-Frequency to High-Frequency heart rate variability (LF/HF) in 10 studies (140 drivers), the root mean square of successive differences among successive R-R normal intervals (RMSSD) in 6 studies (101 drivers), means of R-R intervals (RRI) in 5 studies (46 drivers), the standard deviation of normal R-R intervals (SDNN) in 6 studies (95 drivers), pupil diameter in 3 studies (83 drivers), and trapezius muscle tension in 2 studies (38 drivers). The characteristics of the studies included in the meta-analyses are detailed in Supplementary Material 2.

### Meta-Analyses

The analyses indicated that several physiological measures changed significantly with stress interventions, thereby suggesting a change in drivers' stress state (Table 1). Indeed, heart rate [*g* = 0.42 (0.14 to 0.69), *p* < 0.001] and pupil diameter [*g* = 0.46 (0.02 to 0.90), *p* < 0.05] revealed significant moderate increases, while RRI, a time-domain feature of HRV, indicated a significant moderate decrease [*g* = −0.42 (−0.84 to 0.01), *p* = 0.05] when performing a high-stress driving task compared to a low-stress driving task. In contrast, no significant effects were observed between high-stress and low-stress driving for other measures, including breathing rate [*g* = −0.27 (−0.76 to 0.22), *p* = 0.29], electrodermal activity [*g* = 0.96 (−0.05 to 1.98), *p* = 0.062], LF/HF [*g* = 0.60 (−0.22 to 1.43), *p* = 0.15], RMSSD [*g* = −0.06 (−0.34 to 0.22), *p* = 0.67], SDNN [*g* = −0.19 (−0.47 to 0.10), *p* = 0.20] and trapezius muscle tension [*g* = 0.04 (−0.42 to 0.49), *p* = 0.87]. Among the measures that were found to be significantly sensitive to driver stress, i.e., heart rate, pupil diameter and RRI, none of them showed a real advantage over the others, as indicated by the overlap in their confidence intervals.

**Table 1:** Outcomes of the meta-analyses.

**Physiological measure**	**Sample size**		**Heterogeneity**			**Global effect size**		
	**k**	**N**	***Q*-statistic**	***I^2^*-statistc (%)**	***t* ^2^**	**Hedges' g**	**95%CI**	***p*-value**
Breathing rate	7	156	20.3^**^	66.0	0.23	−0.27	[−0.76; 0.22]	0.29
Electrodermal activity	7	187	67.7^***^	94.0	1.69	0.96	[−0.05, 1.98]	0.062
Heart rate	25	501	127.7^***^	75.3	0.34	0.42	[0.14; 0.69]	<0.001^***^
LF/HF	10	140	71.7^***^	89.6	1.52	0.60	[−0.22; 1.43]	0.15
RMSSD	6	101	0.60	0.00	0.00	−0.06	[−0.34; 0.22]	0.67
RRI	5	46	6.51	0.00	0.00	−0.42	[−0.84; 0.01]	0.05^*^
SDNN	6	95	0.99	0.00	0.00	−0.19	[−0.47; 0.10]	0.20
Pupil diameter	3	83	1.85	20.4	0.038	0.46	[0.02; 0.90]	<0.05^*^
Trapezius muscle tension	2	38	0.11	0.00	0.00	0.04	[−0.42; 0.49]	0.87

*LF/HF, ratio of low frequency to high frequency; RMSSD, root mean square of successive differences among successive R-R normal intervals; RRI, means of R-R intervals; SDNN, standard deviation of normal R-R intervals; k, number of studies; N, number of drivers; Q, I^2^ and τ^2^, statistics used to evaluate heterogeneity of variance; Hedges' g, statistic used to calculate effect size for small sample size; CI, confidence interval; p-value, level of significance*.

* *p < 0.05*,

** *p < 0.01*,

*** *p < 0.001*.

The *Q*-statistics indicated a significant heterogeneity between effect sizes for breathing rate [*Q* = 20.3, *p* < 0.01], electrodermal activity [*Q* = 67.7, *p* < 0.001], heart rate [*Q* = 127.7, *p* value < 0.001] and LF/HF ratio [*Q* = 71.7, *p* value < 0.001]. The degrees of heterogeneity for these measures, subsequently quantified using the *I*^2^-statistic, were found to be moderate to large [Breathing rate: *I*^2^ = 66.0% (16.1 to 94.8); Electrodermal activity: *I*^2^ = 94.0% (84.4 to 98.9); Heart rate: *I*^2^ = 75.3% (61.3 to 90.6); LF/HF: *I*^2^ = 89.6% (77.0 to 96.9)]. Considering, first, the moderate to large degrees of uncertainty of *I*^2^-statistics and, second, the amount of true variance between studies for these measures [Breathing rate: τ^2^ = 0.23; Electrodermal activity: τ^2^ = 1.69; Heart rate: τ^2^ = 0.34; LF/HF: τ^2^ = 1.52], we suspect that a large proportion of the observed variance reflected true heterogeneity.

**Figure 2:**
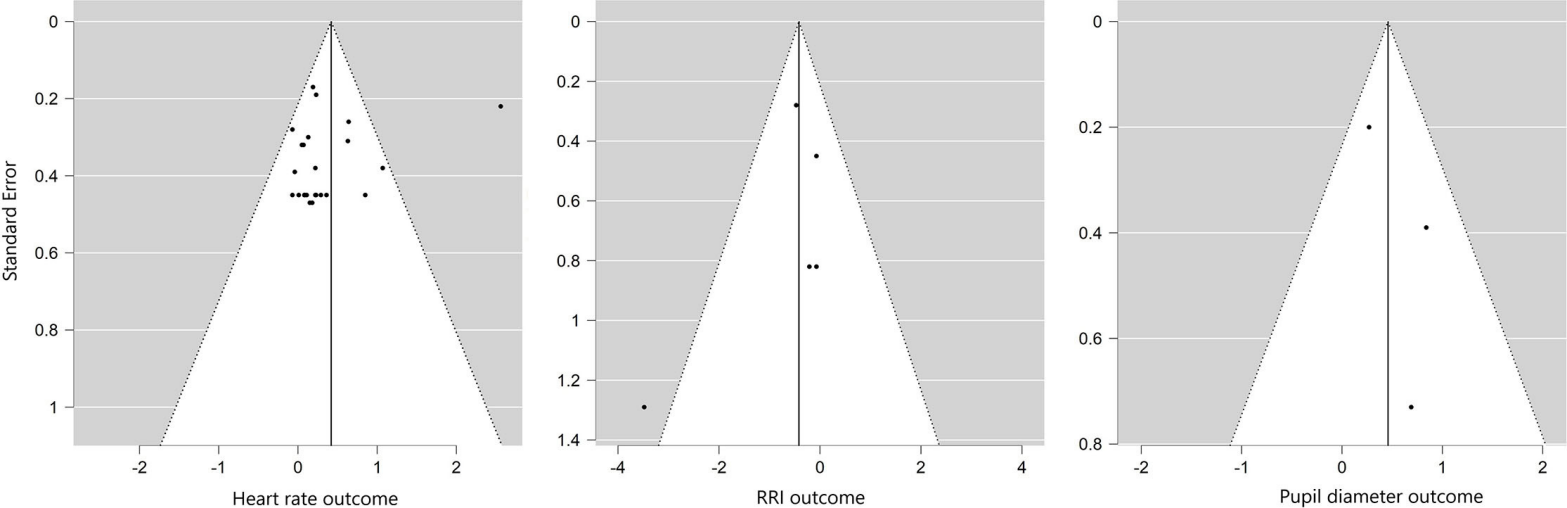
Visualization of funnel plots.

Publication bias investigated by visually inspecting funnel plots for significant measures revealed no asymmetries (Figure 2). The absence of bias was then confirmed by standard rank correlation tests, Egger's regression tests, and fail-safe N analyses [Heart rate: Kendall's τ = 0.25, *p* = 0.10, Egger: *z* = 0.99, *p* = 0.32, Fail-safe N = 350, *p* < 0.001; RRI: Kendall's τ = −0.32, *p* = 0.45, Egger: *z* = −1.26, *p* = 0.21, Fail-safe N = 4, *p* < 0.05; Pupil diameter: Kendall's τ = 0.33, *p* = 1.00, Egger: *z* = 1.05, *p* = 0.29, Fail-safe N = 5, *p* < 0.01].

### Moderator Analyses

To determine the extent to which physiological measures are sensitive to individual and ambient factors, we carried out a series of moderator analyses using subgroups and meta-regressions. Only heart rate met the three eligibility criteria required to conduct moderator analyses: significant summary effect size [*g* = 0.42 (0.14 to 0.69), *p* < 0.001], significant heterogeneity in summary effect size (*Q* = 127.7, *p* < 0.001), and sufficient number of available studies (k = 25 ≥ 5).

### Individual Factors

The moderating effects of age and gender on driver stress were explored (Table 2). Meta-regressions revealed no effect of age (β = −0.015, *p* = 0.22) or gender (β = −0.003, *p* = 0.48).

**Table 2:** Outcomes of individual factors.

**Individual factor**	**physiological measure**	**Sample Size**		**Heterogeneity**			**Global effect size**			
		***k***	***N***	***Q*-statistic**	***I^2^*-statistc (%)**	***t* ^2^**	**Hedges' g**	**β**	**SE**	***p*-value**
**Age**										
	Heart Rate	24	491	114.2^**^	75.1	0.34	0.91	−0.015	0.45	0.22
**Women**										
	Heart Rate	24	491	124.4^**^	76.9	0.36	0.55	−0.003	0.24	0.48

*k, number of studies; N, number of drivers; I^2^, τ^2^ and Q, statistics used to evaluate heterogeneity of variance. Hedges' g, statistic used to calculate effect size for small sample size; β, non-standardized beta coefficient; SE, standard error; p-value, level of significance*.

** *p < 0.01*.

### Ambient Factors

We assessed the moderating effects on driver stress of three ambient factors: apparatus, driving automation and stressor exposure duration (Table 3). The first ambient factor tested was the apparatus. No significant change in heart rate was observed between driving tasks performed in the real-vehicle and driving tasks performed in a driving simulator [*g*_Real_ = 0.37 (0.00 to 0.74), *g*_Simulator_ = 0.41 (0.11 to 0.71)], as revealed by the overlapping of their confidence intervals. These observations were reinforced by the Wald-type test, which did not indicate any significant difference between the two summary effect sizes (*z*_Apparatus_ = 0.44, *p* = 0.66).

**Table 3:** Outcomes of ambient factors.

**Ambient factor**	**Physiological measure**	**Sample size**		**Heterogeneity**			**Global effect size**			
		**k**	**N**	***Q*-statistic**	***I^2^*-statistc (%)**	***t* ^2^**	**Hedges' g**	**95%CI**	**SE**	***p*-value**
**Apparatus**										
Real vehicle	Heart Rate	16	256	97.6^**^	73.4	0.41	0.37	[0.00, 0.74]	0.19	0.053
Driving simulator	Heart Rate	9	245	24.4^**^	57.4	0.11	0.41	[0.11, 0.71]	0.15	<0.01^**^
**Automation**										
Manual	Heart Rate	22	457	124.0^**^	78.0	0.39	0.47	[0.16, 0.77]	0.16	<0.01^**^
Fully autonomous	Heart Rate	3	44	0.2	0.0	0.00	0.09	[−0.33, 0.51]	0.21	0.67
**Duration**										
Short-term	Heart Rate	17	391	108.3^**^	79.9	0.40	0.44	[0.10, 0.79]	0.18	<0.05^*^
Long-term	Heart Rate	8	110	15.1^*^	0.0	7.57e-6	0.22	[−0.05, 0.49]	0.14	0.115

*k, number of studies; N, number of drivers; I^2^, τ^2^ and Q, statistics used to evaluate heterogeneity of variance; Hedges' g, statistic used to calculate effect size for small sample size; CI, confidence interval; SE, standard error; Apparatus, real vehicle vs. driving simulator; Automation, manual vs. autonomous driving; Duration, short-term vs. long-term exposure to the stressor; p-value, level of significance*.

* *p < 0.05*,

** *p < 0.01*.

The second ambient factor we assessed was driving automation. Although heart rate showed a greater overall effect size when stress intervention was performed in manual driving [*g*_Manual_ = 0.47 (0.16 to 0.77)] compared to fully autonomous driving [*g*_Fullyautonomous_ = 0.09 (−0.33 to 0.51)], the overlap in the confidence intervals suggested that the difference was not statistically significant. In addition, the results of the Wald-type test indicated similar summary effect sizes between manual and autonomous driving (*z*_Automation_ = 0.87, *p* = 0.38).

The third ambient factor assessed was the stressor exposure duration. No significant cardiac difference was noticed between short and long-term stress exposure [*g*_short_ = 0.44 (0.10 to 0.79, *g*_Long_ = 0.22 (−0.05 to 0.49)]. The lack of significance was indeed supported by the Wald test result (*z*_Duration_ = 0.31, *p* = 0.76).

## Discussion

To our knowledge, these are the first meta-analyses to investigate (*i*) the sensitivity of each physiological measure in quantifying driver stress, and the moderating effect of (*ii*) population type and (*iii*) driving ambient on driver stress. The main finding is that moderate physiological changes were initiated by stress interventions, suggesting that heart rate, RRI—a time-domain HRV feature—and pupil diameter are sensitive measures for quantifying driver stress. Driver stress indexed by heart rate showed no moderating effect of age, gender, apparatus, driving automation or stressor exposure duration. Below, we provide a summary and interpretations of the results, discuss implications for future research and present the main limitations of the reported work.

### Summary and Interpretation of the Results

Considering the overall effect sizes and their confidence intervals in order to judge the significance of an effect, and thus the sensitivity of a measure, we identified three physiological measures that are sensitive enough to quantify driver stress, namely heart rate, RRI and pupil diameter. The fact that both heart rate and RRI are both sensitive is consistent since heart rate is derived from RRI. It should be noted that of the three sensitive physiological measures (i.e., heart rate, RRI and pupil diameter), none was found to have a significant advantage over any other in identifying driver stress. While these three measures showed sensitivity to driver stress, the other measures did not (i.e., breathing rate, electrodermal activity, LF/HF, RMSSD, SDNN and trapezius muscle tension). However, this does not mean that they are not sensitive. At this stage, we cannot conclude about the lack of sensitivity of these measures. It is indeed possible that the sample size for each of these measures is too small and/or presents too much heterogeneity across studies, which would prevent revealing a sensitivity to driver stress.

Only heart rate warranted moderator analysis because it was the only measure that met all the eligibility criteria. However, individual moderators (age, gender) and ambient moderators (apparatus, driving automation, stressor exposure duration) did not reveal any significant change in heart rate. Despite this, it is very likely that there are moderators of the stress response given the considerable heterogeneity (i.e., high values of Q, *I*^2^ and τ^2^) observed in the effect sizes. Possible explanations regarding the lack of physiological change are provided below.

### Individual Modulators

#### Age and Gender

Although it is well established that individual factors have an impact on stress appraisal (Matthews, 2002), the results regarding the direction of the relationship between individual factors and driver stress have often been contradictory. For example, studies have shown greater stress levels in older populations (Hill and Boyle, 2007), while others have observed lower stress levels in older populations (Langford and Glendon, 2002). Therefore, the aggregation of studies with opposite results in the same meta-analysis could explain our findings about the lack of an age effect on driver stress. Nonetheless, this does not mean that there is no real moderating effect of age. Indeed, the driving experience, closely linked to age (Gulian et al., 1990), can influence the driver stress response, as observed through the stronger correlations between age and all dimensions of driver stress (DBI scales) when driving experience is statistically controlled (Westerman and Haigney, 2000). Also, cognitive decline has been mentioned as a possible explanation for greater stress levels in older populations, which is highlighted, in particular, by a drop in “alertness and anticipation” and an increase in “driving dislike” with age (Westerman and Haigney, 2000). Therefore, the unifactorial approach (i.e., investigating factors one by one) might mask the true effect of moderating factors (e.g., age and gender, lack of experience or negative experiences, awareness of cognitive decline) by not taking account of their interdependence. This is in line with Matthews' (2002) transactional theory of driver stress, according to which driver stress is the result of transactional relationships between several factors.

### Ambient Modulators

#### Apparatus

Although stress studies conducted in a driving simulator offer a more controlled and safe approach, they might nevertheless be poorly representative of the stress experienced under real and ecological conditions. Our results *a priori* seem to contradict this criticism since they suggest that stress induced in a driving simulator and measured by heart rate is indeed representative of stress experienced in real conditions. Indeed, the lack of change in heart rate between driving simulator studies and real vehicle driving studies was observed through similar overall effect sizes, similar standard errors and a non-significant Wald-type test. However, the significant heterogeneity in effect sizes, observed in both simulator and real-road studies, indicates that additional factors explain the overall effect size. We believe that these factors are related to differences in experimental designs, and in particular in the stressful stimuli used. In addition, it cannot be excluded that the nature of the stimuli used and the experimental designs also differ between studies conducted on driving simulators and in real-vehicles. Thus, we can legitimately ask whether the internal driver states we measure in driving simulators and in real road conditions are the same, and if the response to stressful stimuli in real car driving is not shaped by additional safety concerns, among other factors. This is why Milleville-Pennel and Charron (2015) raised the question: “*Can we consider that the same cognitive functions are involved in simulated driving and in real car driving?*.” Furthermore, previous studies have compared internal driver states (not exclusively stress) in simulated and real-world driving using the same stimuli and have measured these states using heart rate (Engström et al., 2005; Johnson et al., 2011; Li et al., 2013; Gemonet et al., 2021). However, no consensus has been reached due to conflicting results. Given both our results and the discrepancy between results in the literature, we recommend further investigating driver stress in both simulated and real vehicle driving using experimental designs that are as similar as possible, i.e., including the same hazardous or stressful stimuli, same driving environment and same participants when doing driving simulator validation studies.

#### Automation

The lack of difference in measures of heart rate between manual and autonomous driving—indicated by a non-significant Wald-type test—indicates *a priori* that driver stress is not influenced by driving automation. Nonetheless, the effect size of stress interventions was significant in manual driving (*g* = 0.47, *p* < 0.01^**^), while it was non-significant in autonomous driving (*g* = 0.09, *p* = 0.67). Taken together, the lack of difference observed between manual and autonomous driving may be due to the small number of included studies that investigated autonomous driving (k = 3). Although no reliable conclusion concerning the possible influence of driving automation on driver stress can be provided at this stage, further investigations of driver stress in autonomous driving are strongly recommended to confirm or refute this lack of effect. In cases where additional studies confirm this lack of effect, it would be interesting to explore the sources. Below, we put forward potential explanations for the lack of an effect of autonomous driving that can be considered as avenues of investigation. First, such a lack of effect may be due to the different nature of the stressors, i.e., more arousing and demanding in terms of cognitive and motor skills for manual driving than for automated driving. Second, it may also be explained by a reduction in driver stress during autonomous driving. This explanation would be consistent with the hypothesis of reduced vulnerability to stress during autonomous driving and related to the decrease in workload (Stanton and Young, 1998, 2005). Third, the lack of effect of stress interventions may also be due to drivers' level of experience with automated driving systems and their trust. As evidence of this, a relationship has previously been found between reported trust in autonomous driving and physiological stress (Morris et al., 2017). Fourth, heart rate may not be a suitable indicator for detecting stress in autonomous driving. Therefore, it would be interesting to consider alternative measures, such as LF/HF ratio (Heikoop et al., 2017) and electrodermal activity (Zontone et al., 2020), both of which have already been used for stress detection purposes during autonomous driving.

#### Duration

The lack of change in heart rate between short-term and long-term driving—highlighted by a non-significant Wald-type test—suggests that the sensitivity of heart rate is not modulated by the stressor exposure duration. However, the effect size of stress interventions was significant in short-term driving (*g* = 0.44, *p* < 0.05^*^), whereas it was non-significant in long-term driving (*g* = 0.22, *p* = 0.115). Although additional studies would be necessary to draw definitive conclusions concerning the existence of cardiac differences depending on the duration of driving under stress conditions, the disparity of the results nevertheless enables us to put forward a first hypothesis. Indeed, it is likely that our findings reflect the effect of the nature of the stressors manipulated within each subgroup (short-term and-long-term) and not the effect of the stressor exposure duration and therefore the measurement time. We believe that event-related and intense stressors are more likely to be studied over short time periods than more diffuse and moderate stressors, which would require longer measures in order to be detected by cardiac sensors. Consequently, in the future, it would be interesting to study the same stressors (i.e., same nature and intensity) while varying only the cardiac measurement time. This would also address the question raised by Antoun et al. (2017) about the existence of a threshold effect beyond which driving in a given context would become significantly more stressful. For exploratory purposes, a driving time cut-off of 10 mins was arbitrarily set when forming the subgroups and it is possible that other values might be more appropriate for highlighting a potential moderating effect of stressor exposure duration on driver stress.

### Implications for Future Research and Practice

Our results aim to shed light on driver stress-sensitive measures in order to assist researchers and practitioners in their measurement decisions. Based on our findings, three physiological measures were found to be sensitive to driver stress, namely heart rate, RRI and pupil diameter. Nonetheless, we recommend that readers interpret our results (i.e., the magnitude of the effects) in the context in which driver stress was manipulated in the included studies. Indeed, as Mehler et al. (2012) suggested, the sensitivity of measures may vary depending on the specific tasks and individual states considered. In addition, we encourage further investigation of the other measures used, which may not have been able to reveal their potential sensitivity in our study, in part because of the limited number of studies and/or failure of studies to meet eligibility criteria.

Considerations for future research and practice arise mainly from the results of sensitivity and moderator analyses. We found, first, that some measures did not exhibit sensitivity to stress and that the studied factors did not highlight a moderating effect on stress despite the large heterogeneity in effect sizes. As a result, we recommend that researchers and practitioners interested in exploring driver stress adopt a 3-step approach in order to optimize the observation of both physiological change reflecting sensitivity and of moderating effects, and, more generally, to improve the understanding of driver stress. The 3-step approach consists of: (1) using multiple measures, (2) combining measurement modalities (triangulation approach), and (3) analyzing how factors (stressors and moderators) interact (multifactorial approach). Below, we advocate these principles for driver stress investigations, although they can also be applied to the exploration of other psycho-physiological and cognitive states.

#### Using Multiple Measures

First, researchers and practitioners should use multiple measures to ensure that the physiological changes induced by stressors are also actually observed. This approach would compensate for the failure of some measures in some individuals or in some study contexts. For example, Healey and Picard (2005) pointed out that the electrodermal response may differ among drivers due to variations in the number of sweat glands on the palms. The question of the reliability of pupil diameter to index driver stress also arises in real road contexts, where the measure can be disturbed by many uncontrollable factors, such as light variation and driver's verbal output (Recarte and Nunes, 2003). According to Mehler et al. (2012), no single physiological measure would provide optimal sensitivity for capturing a given state in all types of tasks. Second, using multiple measures in combination would permit a more reliable identification of driver stress. Indeed, Bernardi et al. (2000) supported the analysis of combined measures after observing the influence of breathing on HRV during simple mental and verbal activities. More specifically in an automotive context, the influence of driver stress resulting from a combination of physiological measures has also been investigated (Ollander et al., 2016). The authors found that combining cardiac, electrodermal and respiratory signals made it possible to distinguish between resting and driving, while combining cardiac and respiratory signals helped distinguish between low-stress driving and high-stress driving (Ollander et al., 2016). Third, the use of multiple measures and features would also provide information about the sympathovagal balance, thus improving knowledge of the psychophysiological mechanisms underlying stress states. Some measures and features reflect the activity of both autonomic components, while others mainly reflect the activity of one of the two components. This knowledge is also particularly interesting for remediation strategies, given that Respiratory Sinus Arrhythmia (RSA) mainly reflects the parasympathetic component (Berntson et al., 1993), that a low RSA and anxiety are related (Thayer et al., 1996) and that it has proved possible to progressively increase RSA using breathing and biofeedback techniques (Climov et al., 2014).

#### Triangulation Approach

In the same way as other works which have previously reviewed studies of stress (Alberdi et al., 2016), and driver stress in particular (Rastgoo et al., 2018; Chung et al., 2019), we advocate the joint use of physiological, subjective, and behavioral measures to explore stress in driving. This approach, also called triangulation (Denzin, 1978), permits the accurate observation of a common phenomenon and enriches its explanation (Jick, 1979). Since such an approach captures the multidimensional responses to stress (Matthews, 2002) at the physiological, behavioral, emotional and cognitive levels, it will help us differentiate between the various stress states experienced by drivers. This will then make it possible to derive stress-sensitive driver profiles (Pesle et al., 2018) and design driver stress detection systems (Rastgoo et al., 2018).

#### Multifactorial Approach

Our results showed no modulating effect of the studied factors (age, gender, apparatus, driving automation, and stressor exposure duration). As suggested above, these findings may be partly due to our univariate approach, which considered each factor independently. This statement is supported by a recent study in which an effect of age on driver stress was found using a multivariate approach (i.e., Principal Component Analysis of physiological measures) (Zhao et al., 2020). This type of approach has been supported by a number of different studies which have observed dependencies between driver stress and various individual and ambient factors, such as personality, mood, coping strategies, age, gender, driving experience, time of day in relation to the circadian rhythms (Langford and Glendon, 2002; Pesle et al., 2018). Our findings, alongside those of previous studies, support the idea that the multivariate approach advocated by Matthews et al. (2017) if we are to achieve a holistic understanding of the moderators (individual and ambient), stressors and outcomes of driving. Nonetheless, this type of approach remains difficult to implement. In this context, the multivariate approach should systematically call on theoretical support, such as the T^2^SO (Time-Trait-Stressors-Outcome) framework proposed by Matthews et al. (2017), to facilitate understanding and test multivariate theories of driver stress. In addition, the use of computational techniques would facilitate the implementation of a multifactorial approach.

## Limitations

### Several Limitations Should be Acknowledged

#### Small Number of Studies

Although the random-effects models used for our meta-analyses were designed to permit us to generalize our results beyond the included studies (Borenstein et al., 2010), the small number of studies nevertheless limits the scope of our interpretations. Given the small number of studies, moderator analyses could be performed for only one stress-sensitive physiological measure; namely, heart rate. Therefore, it cannot be excluded that the results and interpretations of the moderator analyses are dependent on the physiological measure used, in this case heart rate. Interpretations of each moderator are also limited by the small number of studies within some moderator subgroups. This reflects the fact that driver stress has not been sufficiently investigated under specific driving conditions (e.g., autonomous driving). One reason for the small number of studies included in meta-analyses is the exclusion of driver stress studies that used various algorithms to combine physiological signals (Singh et al., 2011; Lanatà et al., 2014; Dobbins and Fairclough, 2018; Bitkina et al., 2019; Hadi et al., 2019). Indeed, we focused on a univariate approach to examine the sensitivity of independent physiological measures. Another major reason is the lack of information about the stress interventions in the studies (e.g., mean and/or standard deviation).

#### Use of Different Stressors

As driver stress has been interpreted in different ways by authors, many stress interventions have been collected across studies (e.g., heavy traffic, complex driving maneuvers, surprising events). Therefore, the effect sizes could be identified more precisely if comparison groups included only highly similar stressors. The wide variety of experimental designs found in the studies did not allow us to achieve such granularity.

#### Highlight Sensitivity of Physiological Measures to Driver Stress, but Not Selectivity (or Specificity)

The current study demonstrated the sensitivity—and not the selectivity—of various physiological measures to driver stress. Sensitivity refers to the capacity of an instrument to detect changes in a given task or situation, whereas selectivity refers to the sensitivity of an instrument only to differences in one state (e.g., stress state) and not changes in other states (e.g., mental workload) (O'Donnell and Eggemeier, 1986; Matthews et al., 2015). It is therefore entirely possible that the physiological measures found to be sensitive to driver stress in this study are also sensitive to other psycho-physiological and cognitive states of the driver. Several factors (i.e., not only stressors) would thus influence the autonomic nervous system responses. Such observations would suggest a lack of selectivity of the physiological measures to driver stress when the measures are used alone and independently, i.e., without combining measures. In favor of this assumption, let's take the example of driver stress-sensitive heart rate. Zontone et al. (2020) noted a systematic difference in heart rate between manual and autonomous driving under all conditions (stress and control), leading them to believe that additional factors, unrelated to stress, were responsible for the changes in heart rate. One of the most likely explanations for these changes in heart rate is the significant influence of motor activity during manual driving. Another possible explanation is that mental workload influences cardiac response, which would consequently be reduced with automation (Stanton and Young, 1998; Young and Stanton, 2002). In addition, Parent et al. (2019) suggested that stress and mental workload would have similar sources and effects. Given these common characteristics, the use of a single physiological measure, in this case heart rate, might be limited in its ability to infer a specific state (e.g., stress state) when several factors interplay (e.g., stress, mental workload, motor activity). The current study investigated the physiological measures alone and independently, therefore it meets the criterion of sensitivity of the physiological measures to driver stress but not selectivity. We believe that the investigation of the selectivity of physiological measures to driver stress can only be done by considering multiple driver states, including multiple measures, combining multiple measurement modalities, and performing an analysis of multiple explanatory factors. Although this approach is highly challenging to implement, we have good reason to believe that the multivariate approach is the key to distinguishing each driver state, including driver stress. In this sense, previous research has shown the specificity of autonomic nervous system responses to basic emotions when these emotions were examined using multivariate analyses (Stephens et al., 2010). Given the importance of emotions (e.g., anger, fear) in the driver's stress response, multivariate analyses might be a powerful tool to enable isolating stress from other psychophysiological and cognitive states. Computational techniques (e.g., preprocessing, feature selection, machine learning) and neuroimaging techniques, which have recently been shown to differentiate stress from workload (Parent et al., 2019), might also contribute to distinguishing all these states.

## Conclusion

This research relied on an empirical approach that aggregates results from the literature to quantify the sensitivity of physiological measures to driver stress. The results showed that heart rate, RRI and pupil diameter were sensitive enough to permit this. We believe that these findings could provide initial support for researchers and practitioners when deciding which physiological measures to use to quantify stress while driving.

Future studies involving these measures, as well as HRV features, electrodermal activity, breathing rate and trapezius muscle tension, are necessary to draw conclusions about their (lack of) sensitivity for quantifying driver stress. Given the growing interest in achieving early detection, we recommend using multiple physiological measures in order to ensure and enhance the observation of stressor-induced physiological changes. Indeed, the design of corrective or assistance solutions that specifically target driver stress and that would be activated as soon as stress emerges would be of interest in terms of safety and comfort. In addition, in order to promote a broad understanding of driver stress involving stressors, modulators and outcomes, we recommend a triangulation-based methodology (using subjective, behavioral and physiological measures) combined with a multifactorial approach (studying several factors simultaneously and jointly). Finally, functional neuroimaging studies should be performed to explore the neurophysiological correlates underlying driver stress states and thus provide additional insights into these states.

## Data Availability Statement

The original contributions presented in the study are included in the article/Supplementary Materials, further inquiries can be directed to the corresponding author.

## Author Contributions

LK, SD, and JN contributed to the meta-analysis process, contributed to manuscript revision, read, approved the submitted version, and collected the data. LK organized the database, wrote the first draft of the manuscript, and performed the statistical analysis. JN revised the statistical analysis. All authors contributed to the article and approved the submitted version.

## Conflict of Interest

The authors declare that the research was conducted in the absence of any commercial or financial relationships that could be construed as a potential conflict of interest.

## Publisher's Note

All claims expressed in this article are solely those of the authors and do not necessarily represent those of their affiliated organizations, or those of the publisher, the editors and the reviewers. Any product that may be evaluated in this article, or claim that may be made by its manufacturer, is not guaranteed or endorsed by the publisher.
