# Supplementary material for: Sensitivity of Physiological Measures of Acute Driver Stress: A Meta-Analytic Review
Source: Front Neuroergon. 2021 Dec 14;2:756473. doi: 10.3389/fnrgo.2021.756473 (PMC10790912; doi:10.3389/fnrgo.2021.756473)
Supplement: Supplementary file 1 [file Table_1.DOCX]

Supplementary Material (S1)

# Formulas

## Computing Cohen’s *d* and and Hedges’ *g* that use independent groups

$$d=\frac{\bar{X}_{1} -\bar{X}_{2}}{\mathrm{sd}_{pooled}}{with sd}_{pooled}=\sqrt{\frac{\left( n_{1}-1 \right){S_{1}}^{2}+\left( n_{2}-1 \right){S_{2}}^{2}}{n_{1}+n_{2}-2}}$$

$\bar{X}_{1}$ and $\bar{X}_{2}$ = sample means in the two groups

$\mathrm{sd}_{pooled}$ = pooled standard deviation of both means

$n_{1}$and $n_{2}$ = sample sizes in each group

$S_{1}$ and $S_{2}$ = standard deviations in each group

$$g=J.d \mathrm{with} J=1- \frac{3}{4.df-1}$$

$J$ = correction factor

$df$ = degrees of freedom (group size minus one)

## Computing Cohen’s *d* and and Hedges’ *g* that use matched groups

$$d=\frac{\bar{Y}_{1} -\bar{Y}_{2}}{\mathrm{sd}_{pooled}}{with sd}_{pooled}=\sqrt{\frac{\left( n_{1}-1 \right){S_{1}}^{2}+\left( n_{2}-1 \right){S_{2}}^{2}}{n_{1}+n_{2}-2}}$$
