# Supplementary material for: Sensitivity of Physiological Measures of Acute Driver Stress: A Meta-Analytic Review
Source: Front Neuroergon. 2021 Dec 14;2:756473. doi: 10.3389/fnrgo.2021.756473 (PMC10790912; doi:10.3389/fnrgo.2021.756473)
Supplement: Supplementary file 2 [file Table_2.DOCX]

Supplementary Material (S2)

# Table S2. The characteristics of the studies included in the meta-analyses in detail.

*Note*. N = number of drivers. Number in square brackets distinguishes different groups of participants investigated in the same study.

| Physiological Measure | Authors | High-Stress Driving | Low-Stress Driving | N | Personal Factor | |  | Ambient Factor | | |  | High-Stress Driving | |  | Low-Stress Driving | |
| --- | --- | --- | --- | --- | --- | --- | --- | --- | --- | --- | --- | --- | --- | --- | --- | --- |
|  |  |  |  |  | Age | Women |  | Apparatus | Automation | Duration |  | Mean | SD |  | Mean | SD |
| Breathing Rate | | | | | | | | | | | | | | | | |
|  | [Haouij et al. (2018)](https://drive.google.com/file/d/1KmUVPqNpKmMgiLA-Jy26CGZWP8QqriPP/view?usp=sharing) | City driving | Highway driving | 9 | 29.9 | 44% |  | Real-vehicle | Manual | Long |  | 18.35 | 2.92 |  | 23.35 | 6.35 |
|  | [Healey & Picard (2005)](https://drive.google.com/file/d/1SqyUW1y7hlZYHxIClnj-EJ5EzcZoKMXX/view?usp=sharing) | City driving | Highway driving | 10 | NA | NA |  | Real-vehicle | Manual | Long |  | 19.06 | 4.48 |  | 19.47 | 3.47 |
|  | [Heikoop et al. (2019)](https://drive.google.com/file/d/1WbRD8wPQBYBi7H9brZ9JaGa24GUn-6ih/view?usp=sharing) | Fully automated driving + Detection task | Fully automated driving + No detection task | 9 | 35.4 | 22% |  | Real-vehicle | Fully autonomous | Long |  | 17.65 | 2.10 |  | 17.24 | 2.11 |
|  | [Napoletano & Rossi (2018)](https://drive.google.com/file/d/1UacxosMdwgvxayLv34zwB8iMtDg4ih-3/view?usp=sharing) | Driving + Stirring questions and texting | Practice and Relaxing driving (following instructions) | 68 | 52.0 | 49% |  | Simulated | Manual | Short |  | 17.02 | 1.29 |  | 17.58 | 0.91 |
|  | [Rendon Velez et al.(2016)](https://drive.google.com/file/d/1QNqpyulzQ4Ci0GmEJ51ERrjmWv7OzGu4/view?usp=sharing) | Urban road driving + Time pressure | Urban road driving + No time pressure | 54 | 27.8 | 15% |  | Simulated | Manual | Short |  | 20.30 | 2.70 |  | 19.00 | 2.65 |
|  | [Y. Zhao et al. (2020) [1]](https://drive.google.com/file/d/1FV6jQjh7azSO0_-6mOtBVpkJpz7TH5w_/view?usp=sharing) | Driving + Turns | Driving + Straight roads | 3 | 22.0 | 0% |  | Real-vehicle | Manual | Short |  | 25.69 | 0.12 |  | 25.98 | 0.15 |
|  | Y. Zhao et al. (2020) [2] | Driving + Turns | Driving + Straight roads | 3 | 62.7 | 0% |  | Real-vehicle | Manual | Short |  | 25.41 | 0.81 |  | 25.96 | 0.14 |
| Electrodermal Activity | | | | | | | | | | | | | | | | |
|  | [Cottrell & Barton (2012)](https://drive.google.com/file/d/1AXf9TQd0KaZgo08stYxLs8NnVWnFQ8Cd/view?usp=sharing) | BEV driving + Manually engaged sounds | BEV driving + No artificial sounds | 72 | 24.0 | 33% |  | Real-vehicle | Manual | Short |  | 0.65 | 0.09 |  | 0.51 | 0.08 |
|  | [Healey & Picard (2005)](https://drive.google.com/file/d/1SqyUW1y7hlZYHxIClnj-EJ5EzcZoKMXX/view?usp=sharing) | City driving | Highway driving | 10 | NA | NA |  | Real-vehicle | Manual | Long |  | 10.92 | 1.94 |  | 9.72 | 1.38 |
|  | [Morris et al. (2017)](https://drive.google.com/file/d/1XdOKbl8Acl5KrB2xubd2CBD0hn9M94a-/view?usp=sharing) | Risky autonomous driving | Safe autonomous driving | 28 | 19.4 | 78% |  | Simulated | Fully autonomous | Short |  | 4.53 | 3.53 |  | 4.21 | 3.48 |
|  | [Paredes et al. (2018)](https://drive.google.com/file/d/1KM_Z-zAsizM99rklAs3HDOC1JS7Q9WSp/view?usp=sharing) | Driving + Turns + Stress stimulus (heavy metal music) | Driving + Turns + Calm stimulus (self-selected music) | 25 | 34.4 | 52% |  | Simulated | Manual | Short |  | 0.37 | 0.47 |  | 0.30 | 0.33 |
|  | [Pedrotti et al. (2014)](https://drive.google.com/file/d/12cYGhrBapqL81x0Bx9bcmPXZ9PlBhg03/view?usp=sharing) | Driving + Sound alerts + 2 human evaluators | Driving + No sounds + No human evaluators | 29 | 41.0 | 51% |  | Simulated | Manual | Short |  | 36.24 | 25.97 |  | 37.77 | 23.56 |
|  | [Ooi et al. (2018)](https://drive.google.com/file/d/1KC9PcKA8eF5H6J7muM3aCG68XlhXgsZL/view?usp=sharing) | Snowy moutain driving (narrow road and poor vision) | Wide circular roadway driving (good vision) | 19 | 22.8 | NA |  | Simulated | Manual | Short |  | 6.34 | 1.15 |  | -1.37 | 2.45 |
|  | [Zontone et al. (2021)](https://drive.google.com/file/d/1d6Pjzbd2paB83u7J9jJCabM2YNP_zF5g/view?usp=sharing) | Oversteering driving | Neutral driving | 4 | NA | 0% |  | Simulated | Manual | Short |  | 0.87 | 0.52 |  | 0.53 | 0.17 |
| Heart Rate | | | | | | | | | | | | | | | | |
|  | [Cottrell & Barton (2012)](https://drive.google.com/file/d/1AXf9TQd0KaZgo08stYxLs8NnVWnFQ8Cd/view?usp=sharing) | BEV driving + Manually engaged sounds | BEV driving + No artificial sounds | 72 | 24.0 | 33% |  | Real-vehicle | Manual | Short |  | 73.63 | 14.34 |  | 44.54 | 7.02 |
|  | [Haouij et al. (2018)](https://drive.google.com/file/d/1KmUVPqNpKmMgiLA-Jy26CGZWP8QqriPP/view?usp=sharing) | City driving | Highway driving | 9 | 29.9 | 44% |  | Real-vehicle | Manual | Long |  | 81.33 | 8.42 |  | 80.01 | 7.82 |
|  | Gotardi et al. (2019) [1] | Competitive environment driving | Quiet environment driving | 16 | 26.4 | 12% |  | Simulated | Manual | Short |  | 92.92 | 14.31 |  | 79.34 | 10.16 |
|  | Gotardi et al. (2019) [2] | Competitive environment driving | Quiet environment driving | 11 | 24.0 | 54% |  | Simulated | Manual | Short |  | 89.9 | 11.92 |  | 79.52 | 10.81 |
|  | [Healey & Picard (2005)](https://drive.google.com/file/d/1SqyUW1y7hlZYHxIClnj-EJ5EzcZoKMXX/view?usp=sharing) | City driving | Highway driving | 10 | NA | NA |  | Real-vehicle | Manual | Long |  | 82.53 | 12.26 |  | 78.19 | 9.84 |
|  | [Heikoop et al. (2017)](https://drive.google.com/file/d/1fDLCviH-Pi7JG_z6aEcwBNx80skEsMe_/view?usp=sharing) | Fully automated driving + Detection task | Fully automated driving + Voluntary task | 22 | 29.6 | 40% |  | Simulated | Fully autonomous | Long |  | 70.9 | 12.50 |  | 69.40 | 10.90 |
|  | [Heikoop et al. (2019)](https://drive.google.com/file/d/1WbRD8wPQBYBi7H9brZ9JaGa24GUn-6ih/view?usp=sharing) | Fully automated driving + Detection task | Fully automated driving + No detection task | 9 | 35.4 | 22% |  | Real-vehicle | Fully autonomous | Long |  | 70.01 | 15.09 |  | 67.49 | 10.33 |
|  | [Khattak et al. (2018)](https://drive.google.com/file/d/1qJuaSdSKNOa3q3zZv2iDrUOxK6q7xXLA/view?usp=sharing) | Driving + No ASCT | Driving + ASCT | 22 | 34.0 | 26% |  | Real-vehicle | Manual | Short |  | 89.20 | 19.04 |  | 79.30 | 10.54 |
|  | [Meesit et al. (2020) [1]](https://drive.google.com/file/d/1QnQBihGq0Wqxjo1lhUOHYIAK4ANpYHnZ/view?usp=sharing) | Highway driving + Narrow raised curb median | Highway driving + Wide raised curb median | 10 | 24.0 | 100% |  | Real-vehicle | Manual | Short |  | 4.65 | 4.92 |  | 3.56 | 4.31 |
|  | Meesit et al. (2020) [2] | Highway driving + Narrow raised curb median | Highway driving + Wide raised curb median | 10 | 24.0 | 0% |  | Real-vehicle | Manual | Short |  | 4.01 | 4.08 |  | 3.06 | 3.75 |
|  | Meesit et al. (2020) [3] | Highway driving + Narrow raised curb median | Highway driving + Wide raised curb median | 10 | 38.0 | 100% |  | Real-vehicle | Manual | Short |  | 3.86 | 2.90 |  | 3.83 | 3.39 |
|  | Meesit et al. (2020) [4] | Highway driving + Narrow raised curb median | Highway driving + Wide raised curb median | 10 | 38.0 | 0% |  | Real-vehicle | Manual | Short |  | 4.47 | 2.38 |  | 4.23 | 2.53 |
|  | Meesit et al. (2020) [5] | Highway driving + Narrow painted median | Highway driving + Wide painted median | 10 | 24.0 | 100% |  | Real-vehicle | Manual | Short |  | 4.10 | 5.29 |  | 4.49 | 5.61 |
|  | Meesit et al. (2020) [6] | Highway driving + Narrow painted median | Highway driving + Wide painted median | 10 | 24.0 | 0% |  | Real-vehicle | Manual | Short |  | 4.57 | 3.93 |  | 4.14 | 5.53 |
|  | Meesit et al. (2020) [7] | Highway driving + Narrow painted median | Highway driving + Wide painted median | 10 | 38.0 | 100% |  | Real-vehicle | Manual | Short |  | 4.15 | 4.63 |  | 2.92 | 3.10 |
|  | Meesit et al. (2020) [8] | Highway driving + Narrow painted median | Highway driving + Wide painted median | 10 | 38.0 | 0% |  | Real-vehicle | Manual | Short |  | 2.67 | 1.42 |  | 2.42 | 2.57 |
|  | [Miller & Boyle (2013) [1]](https://drive.google.com/file/d/1R4IljGB3laKR5gEb6xxW9sYCz94etymv/view?usp=sharing) | Highway driving | Country driving | 20 | 22.0 | 50% |  | Real-vehicle | Manual | Long |  | 71.71 | 0.79 |  | 71.22 | 10.36 |
|  | Miller & Boyle (2013) [2] | Highway driving | Country driving | 20 | 46.2 | 50% |  | Real-vehicle | Manual | Long |  | 77.33 | 0.37 |  | 76.95 | 10.46 |
|  | Miller & Boyle (2013) [3] | Highway driving | Country driving | 14 | 72.9 | 28%% |  | Real-vehicle | Manual | Long |  | 72.00 | 0.18 |  | 70.42 | 9.7 |
|  | [Napoletano & Rossi (2018)](https://drive.google.com/file/d/1UacxosMdwgvxayLv34zwB8iMtDg4ih-3/view?usp=sharing) | Driving + Stirring questions and texting | Practice and Relaxing driving (following instructions) | 68 | 52.0 | 49% |  | Simulated | Manual | Short |  | 77.37 | 2.96 |  | 76.84 | 2.66 |
|  | [Paredes et al. (2018)](https://drive.google.com/file/d/1KM_Z-zAsizM99rklAs3HDOC1JS7Q9WSp/view?usp=sharing) | Driving + Turns + Stress stimulus (heavy metal music) | Driving + Turns + Calm stimulus (self-selected music) | 25 | 34.4 | 52% |  | Simulated | Manual | Short |  | 0.55 | 0.31 |  | 0.57 | 0.29 |
|  | [Rendon Velez et al. (2016)](https://drive.google.com/file/d/1QNqpyulzQ4Ci0GmEJ51ERrjmWv7OzGu4/view?usp=sharing) | Urban road driving + Time pressure | Urban road driving + No time pressure | 54 | 27.8 | 15% |  | Simulated | Manual | Short |  | 82.10 | 12.90 |  | 79.20 | 12.30 |
|  | Schießl (2009) | Driving + Trafic | Driving + No traffic | 6 | 28.0 | 38% |  | Simulated | Manual | Long |  | 0.77 | 0.16 |  | 0.13 | 0.13 |
|  | [X. Zhao et al. (2014)](https://drive.google.com/file/d/1Imefrqvoqxqez2zCBtZXa4eo1Vt7SivO/view?usp=sharing) | Driving + Sharp radius curve | Driving + Large radius curve | 30 | 24.8 | 0% |  | Simulated | Manual | Short |  | 80.59 | 2.54 |  | 79.18 | 1.76 |
|  | [Zontone et al. (2020)](https://drive.google.com/file/d/1nKkiGUmXdLCBw5gZkgZHnu7x3MO4bDXJ/view?usp=sharing) | Autonomous driving + Braking behind truck | Autonomous driving + No braking behind truck | 13 | 31.4 | 39% |  | Simulated | Fully autonomous | Short |  | 75.09 | 8.21 |  | 75.38 | 9.07 |
| LF/HF | | | | | | | | | | | | | | | | |
|  | [Chen et al. (2015)](https://drive.google.com/file/d/1vvEeqy1JcAPX-K7YtvLKQiOMXR7RLshw/view?usp=sharing) | First driving with dangerous scenario (e.g, pedestrian) | Last (ninth) driving with dangerous scenario (e.g, pedestrian) | 30 | 25.5 | 33% |  | Simulated | Manual | Short |  | 6.62 | 1.07 |  | 3.10 | 0.44 |
|  | [Heikoop et al. (2017)](https://drive.google.com/file/d/1fDLCviH-Pi7JG_z6aEcwBNx80skEsMe_/view?usp=sharing) | Fully automated driving + Detection task | Fully automated driving + Voluntary task | 22 | 29.6 | 40% |  | Simulated | Fully autonomous | Long |  | 1.18 | 0.30 |  | 1.07 | 0.30 |
|  | [Heikoop et al. (2019)](https://drive.google.com/file/d/1WbRD8wPQBYBi7H9brZ9JaGa24GUn-6ih/view?usp=sharing) | Fully automated driving + Detection task | Fully automated driving + No detection task | 9 | 35.44 | 22% |  | Real-vehicle | Fully autonomous | Long |  | 1.12 | 0.36 |  | 1.01 | 0.25 |
|  | [Manseer & Riener (2014)](https://drive.google.com/file/d/1kfYDjmLsqPMurZ7QUtSegaKrYGpfyfjz/view?usp=sharing) | Highway driving in tunnels | Highway driving on open road | 9 | 25.3 | 10% |  | Real-vehicle | Manual | Short |  | 5.38 | 2.45 |  | 3.69 | 1.37 |
|  | [Miller & Boyle (2013) [1]](https://drive.google.com/file/d/1R4IljGB3laKR5gEb6xxW9sYCz94etymv/view?usp=sharing) | Highway driving | Country driving | 20 | 22.0 | 50% |  | Real-vehicle | Manual | Long |  | 0.44 | 0,07 |  | 0.44 | 0.33 |
|  | Miller & Boyle (2013) [2] | Highway driving | Country driving | 20 | 46.2 | 50% |  | Real-vehicle | Manual | Long |  | 0.45 | 0.05 |  | 0.37 | 0.41 |
|  | Miller & Boyle (2013) [3] | Highway driving | Country driving | 14 | 72.9 | 28%% |  | Real-vehicle | Manual | Long |  | 0.07 | 0.06 |  | 0.11 | 0.54 |
|  | [Yu et al. (2016)](https://drive.google.com/file/d/1VCswA7zm9zHt0lpkApVd_akM2EMCYZEN/view?usp=sharing) | Normal driving | Waiting for traffic light | 10 | 42.5 | NA |  | Real-vehicle | Manual | Short |  | 0.47 | 0.19 |  | 0.46 | 0.15 |
|  | [Y. Zhao et al. (2020) [1]](https://drive.google.com/file/d/1FV6jQjh7azSO0_-6mOtBVpkJpz7TH5w_/view?usp=sharing) | Driving + Turns | Driving + Straight roads | 3 | 22.0 | 0% |  | Real-vehicle | Manual | Short |  | 0.35 | 0.10 |  | 0.37 | 0.06 |
|  | Y. Zhao et al. (2020) [2] | Driving + Turns | Driving + Straight roads | 3 | 62.7 | 0% |  | Real-vehicle | Manual | Short |  | 0.34 | 0.12 |  | 0.32 | 0.10 |
| RMSSD | | | | | | | | | | | | | | | | |
|  | [Miller & Boyle (2013) [1]](https://drive.google.com/file/d/1R4IljGB3laKR5gEb6xxW9sYCz94etymv/view?usp=sharing) | Highway driving | Country driving | 20 | 22.0 | 50% |  | Real-vehicle | Manual | Long |  | 43.52 | 0.86 |  | 45.81 | 16.99 |
|  | Miller & Boyle (2013) [2] | Highway driving | Country driving | 20 | 46.2 | 50% |  | Real-vehicle | Manual | Long |  | 35.83 | 3.74 |  | 35.44 | 38.62 |
|  | Miller & Boyle (2013) [3] | Highway driving | Country driving | 14 | 72.9 | 28%% |  | Real-vehicle | Manual | Long |  | 87.00 | 9.09 |  | 87.62 | 115.89 |
|  | [Paredes et al. (2018)](https://drive.google.com/file/d/1KM_Z-zAsizM99rklAs3HDOC1JS7Q9WSp/view?usp=sharing) | Driving + Turns + Stress stimulus (heavy metal music) | Driving + Turns + Calm stimulus (self-selected music) | 25 | 34.4 | 52% |  | Simulated | Manual | Short |  | 0.34 | 0.30 |  | 0.32 | 0.34 |
|  | [Tavakoli et al. (2020)](https://drive.google.com/file/d/1dkKUllqrFAg0J9oeGc5gFMgmShK0INWS/view?usp=sharing) | City driving | Highway driving | 12 | 27.7 | 50% |  | Real-vehicle | Manual | Long |  | 9.30 | 4.84 |  | 10.40 | 5.19 |
|  | [Yu et al. (2016)](https://drive.google.com/file/d/1VCswA7zm9zHt0lpkApVd_akM2EMCYZEN/view?usp=sharing) | Normal driving | Waiting for traffic light | 10 | 42.5 | NA |  | Real-vehicle | Manual | Short |  | 17.32 | 9.61 |  | 19.01 | 11.08 |
| RRI | | | | | | | | | | | | | | | | |
|  | [Magana & Munos-Organero (2017)](https://drive.google.com/file/d/1vNT7Y23DHe_N6HH-DJ7qsWpRC5xtja0J/view?usp=sharing) | Driving + Rush hour + Go to work | Driving + Calm hour + No go to work | 3 | 39.5 | NA |  | Real-vehicle | Manual | Long |  | 622.80 | 16.41 |  | 737.03 | 33.21 |
|  | [Rastgoo et al. (2019)](https://drive.google.com/file/d/1LmNx6REHmxSW7swOz0oBrJsWqMKpDGTC/view?usp=sharing) | City driving | Highway driving | 27 | 30.5 | 45% |  | Simulated | Manual | Short |  | 790.00 | 53.94 |  | 814.31 | 47.82 |
|  | [Yu et al. (2016)](https://drive.google.com/file/d/1VCswA7zm9zHt0lpkApVd_akM2EMCYZEN/view?usp=sharing) | Normal driving | Waiting for traffic light | 10 | 42.5 | NA |  | Real-vehicle | Manual | Short |  | 772.50 | 154.70 |  | 784.60 | 150.40 |
|  | [Y. Zhao et al. (2020) [1]](https://drive.google.com/file/d/1FV6jQjh7azSO0_-6mOtBVpkJpz7TH5w_/view?usp=sharing) | Driving + Turns | Driving + Straight roads | 3 | 22.0 | 0% |  | Real-vehicle | Manual | Short |  | 0.86 | 0.13 |  | 0.89 | 0.15 |
|  | Y. Zhao et al. (2020) [2] | Driving + Turns | Driving + Straight roads | 3 | 62.7 | 0% |  | Real-vehicle | Manual | Short |  | 1.36 | 0.71 |  | 1.42 | 0.62 |
| SDNN | | | | | | | | | | | | | | | | |
|  | [Heikoop et al. (2017)](https://drive.google.com/file/d/1fDLCviH-Pi7JG_z6aEcwBNx80skEsMe_/view?usp=sharing) | Fully automated driving + Detection task | Fully automated driving + Voluntary task | 22 | 29.6 | 40% |  | Simulated | Fully autonomous | Long |  | 75.6 | 29.50 |  | 75.50 | 30.70 |
|  | [Heikoop et al. (2019)](https://drive.google.com/file/d/1WbRD8wPQBYBi7H9brZ9JaGa24GUn-6ih/view?usp=sharing) | Fully automated driving + Detection task | Fully automated driving + No detection task | 9 | 35.44 | 22% |  | Real-vehicle | Fully autonomous | Long |  | 49.79 | 21.82 |  | 51.51 | 17.53 |
|  | [Miller & Boyle (2013) [1]](https://drive.google.com/file/d/1R4IljGB3laKR5gEb6xxW9sYCz94etymv/view?usp=sharing) | Highway driving | Country driving | 20 | 22.0 | 50% |  | Real-vehicle | Manual | Long |  | 65.14 | 2.68 |  | 68.92 | 15.23 |
|  | Miller & Boyle (2013) [2] | Highway driving | Country driving | 20 | 46.2 | 50% |  | Real-vehicle | Manual | Long |  | 43.33 | 0.64 |  | 48.36 | 29.4 |
|  | Miller & Boyle (2013) [3] | Highway driving | Country driving | 14 | 72.9 | 28%% |  | Real-vehicle | Manual | Long |  | 68.66 | 3.17 |  | 71.87 | 67.8 |
|  | [Yu et al. (2016)](https://drive.google.com/file/d/1VCswA7zm9zHt0lpkApVd_akM2EMCYZEN/view?usp=sharing) | Normal driving | Waiting for traffic light | 10 | 42.5 | NA |  | Real-vehicle | Manual | Short |  | 22.01 | 9.41 |  | 25.80 | 9.68 |
| Pupil Diameter | | | | | | | | | | | | | | | | |
|  | [Pedrotti et al. (2014)](https://drive.google.com/file/d/12cYGhrBapqL81x0Bx9bcmPXZ9PlBhg03/view?usp=sharing) | Driving + Sound alerts + 2 human evaluators | Driving + No sounds + No human evaluators | 29 | 41.0 | 51% |  | Simulated | Manual | Short |  | 0.09 | 0.26 |  | -0.16 | 0.33 |
|  | [Rendon Velez et al.(2016)](https://drive.google.com/file/d/1QNqpyulzQ4Ci0GmEJ51ERrjmWv7OzGu4/view?usp=sharing) | Urban road driving + Time pressure | Urban road driving + No time pressure | 50 | 27.8 | 15% |  | Simulated | Manual | Short |  | 5.37 | 0.66 |  | 5.19 | 0.68 |
|  | [Zontone et al. (2021)](https://drive.google.com/file/d/1d6Pjzbd2paB83u7J9jJCabM2YNP_zF5g/view?usp=sharing) | Oversteering driving | Neutral driving | 4 | NA | 0% |  | Simulated | Manual | Short |  | 0.95 | 0.00 |  | 0.95 | 0.01 |
| Trapezius Muscle Tension | | | | | | | | | | | | | | | | |
|  | [Healey & Picard (2005)](https://drive.google.com/file/d/1SqyUW1y7hlZYHxIClnj-EJ5EzcZoKMXX/view?usp=sharing) | City driving | Highway driving | 10 | NA | NA |  | Real-vehicle | Manual | Long |  | 1.69 | 1.85 |  | 1.40 | 1.32 |
|  | [Morris et al. (2017)](https://drive.google.com/file/d/1XdOKbl8Acl5KrB2xubd2CBD0hn9M94a-/view?usp=sharing) | Risky autonomous driving | Safe autonomous driving | 28 | 19.4 | 78% |  | Simulated | Fully autonomous | Short |  | 11.10 | 31.21 |  | 11.42 | 33.42 |
